# Exercise efficacy and prescription during treatment for pancreatic ductal adenocarcinoma: a systematic review

**DOI:** 10.1186/s12885-020-07733-0

**Published:** 2021-01-09

**Authors:** Dominic O’Connor, Malcolm Brown, Martin Eatock, Richard C. Turkington, Gillian Prue

**Affiliations:** 1 School of Nursing and Midwifery, Queen’s University, 8 Fitzwilliam Street, Belfast, Northern Ireland BT9 6AW UK; 2 The Northern Ireland Cancer Centre, Belfast City Hospital, Belfast, Northern Ireland; 3 The Patrick G Johnston Centre for Cancer Research, Queen’s University, Belfast, Northern Ireland

## Abstract

**Background:**

Surgical resection remains the only curative treatment for pancreatic cancer and is associated with significant post-operative morbidity and mortality. Patients eligible for surgery, increasingly receive neo-adjuvant therapy before surgery or adjuvant therapy afterward, inherently exposing them to toxicity. As such, optimizing physical function through exercise during treatment remains imperative to optimize quality of life either before surgery or during rehabilitation. However, current exercise efficacy and prescription in pancreatic cancer is unknown. Therefore, this study aims to summarise the published literature on exercise studies conducted in patients with pancreatic cancer undergoing treatment with a focus on determining the current prescription and progression patterns being used in this population.

**Methods:**

A systematic review of four databases identified studies evaluating the effects of exercise on aerobic fitness, muscle strength, physical function, body composition, fatigue and quality of life in participants with pancreatic cancer undergoing treatment, published up to 24 July 2020. Two reviewers independently reviewed and appraised the methodological quality of each study.

**Results:**

Twelve studies with a total of 300 participants were included. Heterogeneity of the literature prevented meta-analysis. Exercise was associated with improvements in outcomes; however, study quality was variable with the majority of studies receiving a weak rating.

**Conclusions:**

High quality evidence regarding the efficacy and prescription of exercise in pancreatic cancer is lacking. Well-designed trials, which have received feedback and input from key stakeholders prior to implementation, are required to examine the impact of exercise in pancreatic cancer on key cancer related health outcomes.

**Supplementary Information:**

The online version contains supplementary material available at 10.1186/s12885-020-07733-0.

## Background

In 2018, 458,918 new cases of pancreatic cancer were reported worldwide [1]. Of all pancreatic cancer neoplasms diagnosed, pancreatic ductal adenocarcinoma (PDAC) accounts for more than 90% [2]. Surgical resection remains the only potentially curative treatment for PDAC; however, only 10–20% of individuals have clearly resectable disease at time of diagnosis [3]. For those with resectable disease, surgery is associated with a high risk of post-operative morbidity and surgery alone is associated with poor median and 5-year survival rates of 15–20 months and 8–15% respectively [4]. As such, the addition of adjuvant chemotherapy has become standard of care in an attempt to prolong survival [5]. In addition, neo-adjuvant therapy may increase the resectability of borderline resectable disease and presents advantages in tumour control, higher R0 resection rate,better patient selection (surgery avoided in those whose disease progresses with neo-adjuvant therapy), and improved survival outcomes [6, 7]. However, common treatments are associated with chronic toxicities including heightened fatigue and pain, weight loss and psychological impairment [8]. Furthermore, cancer cachexia is observed in > 85% of individuals with pancreatic cancer at time of diagnosis and is associated with impaired mobility, morbidity and reduced survival [5, 9, 10]. The toxicities of pancreatic cancer treatment are associated with impaired physical function and health related quality of life (HRQoL) and may contribute to greater morbidity and mortality in these patients. As such, there is a need for adjunct therapies to counteract these treatment complications.

Lifetime physical activity is fundamental to health and quality of life [11]. Conventional exercise including aerobic and strengthening exercises, carried out during cancer treatment may help mitigate many of the treatment / disease associated adverse complications. Indeed, exercise is recommended across the cancer care continuum with strong evidence supporting its role in targeting cancer related health outcomes including fatigue, pain and maintaining / improving / restoring physical function [12]. However, the vast majority of high-quality studies from which current exercise guidelines are based, predominantly included participants with early stage breast cancer [13]. In general, these individuals are healthier and more active than the wider cancer population [14] and particularly more than individuals with pancreatic cancer who are mostly diagnosed in the advanced stages of the disease [15]. This makes exercise recommendations from early stage breast cancer potentially unrealistic and unachievable for this group. As such, due to this and the lower prevalence of pancreatic cancer, the feasibility and current exercise efficacy and prescription is unknown. The purpose of this review is to summarise the published literature on exercise studies conducted in patients with pancreatic cancer undergoing treatment with a focus on determining the current prescription and progression patterns being used in this population. This will help in the development of future exercise interventions and guide clinical practice.

## Methods

A systematic search of four databases was conducted using the PRISMA guidelines. Databases, which were searched up to 24 July 2020, included Medline, EMBASE, CINAHL and the Cochrane database. The search strategy included MeSH terms and free keywords as follows: ((Pancrea* cancer OR Carcinoma, pancreatic ductal) AND (Exercise OR rehabilitation OR prehabilitation) AND (fitness OR physical function OR quality of life)). We also examined the reference lists of retrieved original and review articles. A protocol detailing the planned search strategy and method for analysis for this review was registered online with PROSPERO, a register of systematic reviews (CRD42020172234).

### Eligibility criteria

Selection criteria for inclusion in this review comprised; 1) article or abstract of original research, 2) population of pancreatic cancer patients, 3) interventions detailing exercise training (aerobic and / or resistance exercise), and 4) measurement of outcomes pre-exercise and post-exercise to evaluate treatment effectiveness. No limitations were placed by study methodology to allow for a comprehensive overview of the area. Exclusion criteria included pre-clinical studies.

### Search

A search strategy **(**Additional file 1**)** was based on the PICO method and guided by an institutional liaison librarian. The population of interest was individuals with a PDAC diagnosis undergoing systemic therapy (neo-adjuvant, adjuvant), the intervention was conventional exercise (i.e aerobic and muscle strengthening), the comparator was standard / usual care or no intervention, and outcomes of interest were peak / maximal oxygen consumption (VO_2peak_ / VO_2max_) or submaximal exercise capacity, muscle strength, body composition, fatigue and quality of life.

### Study selection

Studies were screened by title, by one reviewer (DO’C) after removal of duplicates. Studies were independently screened by abstract and full text by two reviewers (DO’C, GP). Disagreements were resolved through discussion, and when agreement was not reached, a third reviewer (MB) acted as arbiter. Reasons for exclusion were reported.

### Data collection process

One reviewer (DO’C) extracted data variables: study type, diagnosis, age, treatment, intervention, outcome measures and results **(**Table 1). In addition, exercise intervention prescription and progression data were extracted using frequency, intensity, time, type, volume and progression (FITT-VP), along with safety / adverse event data **(**Table 2**)**.

**Table 1:** Summary of included studies

Author (date)	Study type	Diagnosis	Age (y)	Treatment	Intervention	Control/ comparison	Outcomes	Results
Banzer et al. (2014)	Single-arm prospective	Stage I-IV pancreatic cancer (n = 3)	Range: 54–65 years	Adjuvant chemotherapy w/ Gemcitabine	Home-based aerobic exercise	N/A	Measured pre and post intervention Aerobic capacity (CPET-VO_2peak_) Quality of life (EORTC) Fatigue (EORTC Fatigue symptom subscale)	VO_2peak_ Participant 1: + 5.7 ml/kg/min^− 1^ Participant 2: + 8.7 ml/kg/min^− 1^ Participant 3: − 3.2 ml/kg/min^− 1^ Quality of life (point change score) Participant 1: 0 Participant 2: + 25 Participant 3: + 17 Fatigue symptom scale Participant 1: + 22 Participant 2: + 11 Participant 3: + 11
Cormie et al. (2014)	Case report	invasive colloid adenocarcinoma T2 N1 M0 stage IIb	49 years	Surgery > adjuvant chemotherapy and radiotherapy	Supervised exercise (aerobic /resistance exercise) 3 months post-surgery	N/A	Measures at baseline, 3, 6 months 400 m walk, 1RM leg press, 5xSTS, stair climb, usual / fast paced − 6 m walk, static balance Body composition, BMD (DXA) PA levels (GLTEQ) Quality of life (SF-36, FACT-Hep) Fatigue (FACT-fatigue)	Adherence: 35 / 48 sessions completed (73%) Change from baseline- 400 m walk time (s) at 3 months: − 5.9%, at 6 months: − 17.5% Leg press 1RM (kg) 3 months: + 31.6%, 6 months: + 42.1% 5xSTS (s) 3 months: − 17.2%, 6 months: − 28.2% Stair climb (s) 3 months: − 9.9%, 6 months: − 19.1% Whole body lean mass (kg) 3 months: + 2.9%, 6 months + 3.3% Appendicular lean mass (kg) 3 months: + 3.4%, 6 months: + 8.2% Lumbar spine BMD (g.cm^− 2^) 3 months: − 0.5%, 6 months: + 0.4% Quality of life: S-36 Improved subscales at 3 months: range 19–61%, 6 months: 34–150% FACT-Hep improved subscales at 3 months: range 20–109%, 6 months: 15–127% Fatigue Improved at 3 months: 350%, 6 months: 488%
Marker et al. (2018)	Case series	Recently diagnosed (< 4 weeks) borderline-resectable pancreatic adenocarcinoma (*n* = 3)	Range: 70–74 years	Neo-adjuvant chemotherapy	Supervised, tailored exercise (aerobic/resistance/flexibility) for duration of neo-adjuvant therapy	No control	Measures at baseline, 2 wks preoperatively, 6wks post discharge (* participant 3 received no surgery, post intervention only) Body composition, 400 m walk, fast gait speed, usual gait speed, 30STS, HGS, stair climb Quality of life (FACT-G) Fatigue (FACIT-F)	Lean body mass change from baseline, Participant 1: at pre-op + 15%, at follow-up + 3 Participant 2: at pre-op + 1%, at follow-up − 6% *Participant 3: post intervention + 4% 400-m walk Participant 1: at pre-op + 11%, follow up + 8% Participant 2: pre-op − 4%, follow up 0% Participant 3: post intervention + 11% USG Participant 1: pre-op − 8%, follow-up 0% Participant 2: pre-op + 7%, follow-up + 13% Participant 3: post intervention − 6% FSG Participant 1: pre-op 0%, follow-up 0% Participant 2: pre-op + 4%, follow-up − 22% Participant 3: post intervention + 9% Stair climb Participant 1: pre-op − 11%, follow-up − 14% Participant 2: pre-op − 21%, follow-up − 28% Participant 3: post intervention + 5% HGS Participant 1: pre-op + 3% (D), − 8% (ND), follow-up − 2% (D) -1% (ND) Participant 2: pre-op + 2% (D), − 7% (ND), follow-up − 12% (D), − 23% (ND) Participant 3: post intervention − 1% (D), − 4% (ND) 30STS Participant 1: pre-op + 54, follow up + 8% Participant 2: pre-op + 44% follow-up + 11 Participant 3: post intervention 0%
McLaughlin et al. (2019)	Case report	locally advanced pancreatic adenocarcinoma with invasion of the superior mesenteric vein stage III	47	Adjuvant chemotherapy w/ Folfirinox	Supervised exercise (aerobic/resistance exercise)	N/A	Measured at baseline, 4, 8, 12-week Aerobic capacity (estimated VO2max) Lower / upper body strength (12-RM) Flexibility: seated toe-reach Function: 5xSTS, usual/fast/backwards 6-m walk Body composition (BIA) Quality of life (FACT-Hep) Fatigue (FACIT-fatigue)	Adherence 94% (15/16) Results reported as improvement from baseline at 4, 8, 12 weeks, but not explicitly stated. Figures are estimated from study graphs Estimated VO2max: + 6%, + 8%, + 8% All strength measures improved from baseline at each time point Flexibility: not reported 5xSTS: + 2%, + 17%, + 8% 6-m walk: + 17%, + 7%, + 15% Body composition reported as change from baseline to 12 weeks Body fat % -4.4 Lean mass % + 4.3 Quality of life: + 42%, + 40%, + 38% Fatigue: + 78%, + 84%, + 114%
Mouri et al. (2018)	Single-arm prospective	Stage III and IV pancreatic cancer (*n* = 6)	74 ± 3	gemcitabine plus nab-paclitaxel	Home-based resistance training	No control	Quality of life (EORTC)	Global QOL score: T1–56 ± 37, T2–60 ± 32, T3–55 ± 34 Physical subscale QoL: T1–82 ± 21, T2–85 ± 20, T3–75 ± 28
Naito et al. (2018)	Single-arm prospective	Stage III and IV pancreatic cancer (n = 6)	74 ± 3	gemcitabine plus nab-paclitaxel	Home-based resistance training	No control	6 min walk test 5 m gait speed 5xSTS Hand grip strength	6MWT: T1–459 ± 56 m, T2 – N/R, T3–477 ± 51 m 5 m gait speed: T1–1.2 ± 0.2 m/s, T2 – N/R, T3–1.1 ± 0.3 m/s 5xSTS: T1–11 ± 1, T2–10 ± 3, T3–13 ± 10 Hand grip strength: T1–23.7 ± 4.3 kg, T2–24.0 ± 5.1 kg, T3–22.3 ± 4.6 kg
Ngo-Huang et al. (2017)	Single-arm prospective	Resectable pancreatic adenocarcinoma (*n* = 15)	Mean: 66 ± 6	Chemotherapy and/or chemoradiation	Pre-operative aerobic and resistance exercise (home-based, unsupervised)	No control	Measures at baseline (*n* = 20), 1-week pre-surgery (n = 15), 4 weeks post-surgery Primary outcome: Adherence-Self-report exercise minutes (IPAQ) Secondary outcomes: 10-m walk Dynamic gait index (Balance) 5 x STS (strength) Self-report physical function (PROMIS-sf)	12/15 met aerobic exercise recommendation 6/15 met resistance exercise recommendation 11/15 met or exceeded weekly exercise recommendation (120mins) Pre-operative: 98.6 ± 69.8 mins (aerobic), 57.4 ± 36 min (resistance) Mean: 156.0 ± 64.5 weekly total exercise During chemoradiation aerobic (128.6 ± 106 vs 48.0 ± 35.3 resistance, *p* = .04 PROMIS declined baseline to post-operative *p* = .03 Grip strength decline pre-op to post op, *p* = .03 No other changes in secondary measures
Ngo-Huang et al. (2019)	Single-arm prospective	Resectable pancreatic adenocarcinoma (*n* = 45)	Mean: 66 ± 8	Surgery + neo-adjuvant chemo-radiotherapy	Pre-operative aerobic and resistance exercise (home-based, unsupervised)	No control	Measures at baseline and follow-up 6MWT, 5xSTS, HGS, 3-m walk FACT-Hep, FACT-G	48% underwent curative surgery Change form baseline to follow-up 6MWT + 26 m, *p* = .001 5xSTS 0.8 s, *p* = .049 3-m walk + 0.5 m.^s^, *p* = .009 No change in HGS, *p* = .90 No significant changes in QoL outcomes, *p* = .09
Niels et al. (2018)	Case report	Stage IV pancreatic carcinoma in tail (peritoneal metastases)	46 yr old	Palliative, neo-adjuvant, surgery, adjuvant	Supervised concurrent exercise	N/A	Measures at baseline, 3 months and 7 months Leg extension, curl, chest press, row, back extension, ab crunch, 30/15 W cross walker and bicycle test EORTC, HADS Physical activity levels (GPAQ)	Progressed - palliative therapy > neo-adjuvant therapy >surgery > adjuvant Body weight maintained during neo-adjuvant chemotherapy. All functional outcomes improved at 3 months Performed watt + 39% endurance exercise Seated row + 9 Leg extension + 79% Chest press + 38% Global QoL + 16.6% All functional outcomes improved at 7 months, with the exception of: Abdominal crunch − 88.4% Leg curl − 3.6%
Stiendorf et al. (2019)	3 arm RCT	Resectable or non-resectable PDAC (I-IV) (*n* = 47)	Mean: 60.5 ± 8.4	neo-adjuvant chemotherapy	Home-based RT and/vs supervised RT	Usual care	Measures: baseline (*n* = 65), 3 months (*n* = 55), 6 months (n = 47) Quality of life (EORTC + PAN26 module) Fatigue (MFI)	No change in quality of life or fatigue outcomes at 6 months When resistance exercise groups pooled, mean group difference at 3 months for Global quality of life (*p* = 0.016), physical functioning (*p* = 0.016), cognitive functioning (*p* = 0.008) and sleep problems (*p* = 0.016) were all significantly different. Similar results reported for Physical fatigue subscale (*p* = 0.019), reduced activity (*p* = 0.018) and reduced motivation (*p* = 0.028)
Wiskemann et al. (2019)	3 arm RCT	Resectable or non-resectable PDAC (I-IV) (*n* = 43)	Supervised: 62.8 (6.4) Home-based: 61.1 (8.7) UC: 57.8 (8.2)	neo-adjuvant chemotherapy	Home-based RT and/vs supervised RT	Usual care	Measures at: baseline (*n* = 65) 6 months (*n* = 43) Adherence (self-report logs) Strength (isokinetic), HGS CPET, 6MWT,	Mean overall adherence was 59.2% MIPT: RT1 vs CON elbow flexors (*p* = 0.02) extensors (*p* = 0.01) but not lower limb. RT1 vs RT2 elbow flexors and extensor (*p* < 0.05) MVIC: RT1 vs CON elbow flexors (p = 0.02) knee extensors (p = 0.01). RT2 vs CON knee extensors (*p* = 0.05 RT1 vs RT2 no difference CPET: RT1 vs CON / RT2 peak work rate (both p < 0.05) VO2peak (L/min) RT1 vs RT2 *p* > 0.05, RT1 vs CON, p > 0.05, RT2 vs CON, p > 0.05 Body weight: + 3.2% RT1, − 0.4% RT2. Weight loss > 5% observed in *n* = 14 over intervention
Yeo et al. (2012)	2 arm prospective RCT (*n* = 102)	Resected pancreatic and periampullary cancer (n = 102)	Mean: IG: 66 (38–87) UCG: 67 (48–91)	Adjuvant chemotherapy	Home-based walking	Usual care	Measures: baseline, post intervention (3–6 months) Fatigue (FACIT) Pain (VAS) Observed walk (distance / time) Self-report diary (monthly) General health (SF-36v2) ECOG	Walking distance; IG 2 miles, vs 1 mile UCG (*p* = 0.1) IG sig more likely to be walking / active (80 v 58%, *p* = 0.04) At baseline, mod-severe fatigue in 85% of participants Baseline fatigue not different between groups (mean 27 vs 30). At POST, IG group fatigue better (p = 0.05) Pain (mild in both groups at baseline, VAS = 2.9). improved in both groups POST (1.6 & 1.8) ECOG scores fell in IG (1.6–1.5), increased in UCG (1.5–1.8) SF-36 health survey, 6 of 8 domains improved in IG, 4 of 8 UCG Mental and physical components both improved IG, MCS in UCG

*BIA* bioelectrical impedance analysis, *BMD* bone mineral density, *CPET* cardiopulmonary exercise test, *DXA* dual-energy x-ray absorptiometry *ECOG* Eastern Cooperative Oncology Group, *EORTC QLQ C30 / PAN26* European Organization for Research and Treatment of Cancer Quality of Life Core 30 / pancreatic cancer specific questionnaire, *FACT* Functional Assessment of Cancer Therapy, *FACIT* Functional Assessment of Chronic Illness Therapy-Fatigue, *HADS* (Hospital Anxiety and Depression Scale, *HGS* hand grip strength IG – intervention group, *MFI* Multi-dimensional Fatigue Inventory *MIPT* maximum isokinetic peak torque, *MVIC* maximum isometric voluntary contraction *N/R* not recorded, *VAS UC* usual care, *UCG* usual care group, *FSG*– fast speed gait, *USG* usual speed gait, *MCS* mental component summary, *STS* sit to stand, *VAS* visual analogue scale, (* participant 3 received no surgery, post intervention only)

**Table 2:** Summary of exercise prescritption and progression patterns

Author/ date	Frequency of exercise prescription	Intensity of exercise prescription	Time of exercise session	Duration of exercise intervention	Type of exercise prescribed	Exercise intervention setting	Progression patterns documented	Adverse events / safety
Banzer et al. (2014)	3–5 x/week	Moderate (RPE 13–14)	30–45 min	16 weeks	Hiking, walking, running, cycling, swimming	Home based with option of supervised exercise (Nordic walking) 1x/week	Exercise prescription adjusted to their condition, side-effect status and exercise readiness after 4 weeks No other details provided	Not reported
Cormie et al. (2014)	Twice weekly	Aerobic: 65–80% HRmax or RPE 11–13 (Borg 6–20) Resistance: Moderate to high (2–4 sets, 6–12-RM)	Aerobic: 15–20 min	6 months	Aerobic (walking or cycling) and resistance (10 exercises, machine based, upper and lower muscle groups)	Supervised, exercise clinic	2 one-on-one sessions, followed by group exercise	No adverse events reported
Marker et al. (2018)	2–3 x/week	Aerobic: < 85% HRmax Resistance: RPE > 7 (Borg 0–10)	60 mins	17–21 weeks	Aerobic (walking, cycling or rowing) and resistance (body weight, machine based, free weights, upper and lower muscle groups)	Supervised, unspecified setting	No progression described	Not reported
McLaughlin et al. (2019)	Twice weekly	Aerobic: 70% HRmax Resistance: 3 sets, 12 reps, 60% 1-RM	Aerobic: 15 mins	12 weeks	Aerobic (cycling) and resistance (8 exercises, machine based, lower muscle groups only – PICC)	Supervised	No progression described	No adverse events reported
Mouri et al. (2018)	Daily	Low intensity, 3 sets, 10 reps	30 mins	8 weeks	Resistance exercise	Unsupervised, home-based	Intervention modified by study instructor according to performance and tolerability as identified by self-report diary and interview Self-modification recommended, based on participants feelings of nausea / fatigue	Adverse events reported in *n* = 5. Muscle pain (n = 2), arthralgia (n = 1), dyspnoea on exertion (*n* = 1) plantar aponeurosis (n = 1)
Naito et al. (2018)	Daily	Low intensity, 3 sets, 10 reps	30 mins	8 weeks	Home-based resistance exercise	Unsupervised, home-based	Intervention modified by study instructor according to performance and tolerability as identified by self-report diary and interview Self-modification recommended, based on participants feelings of nausea / fatigue	Adverse events reported in *n* = 5. Muscle pain (*n* = 2), arthralgia (n = 1), dyspnoea on exertion (*n* = 1) plantar aponeurosis (*n* = 1)
Ngo-Huang et al. (2017)	Aerobic: 3x/week Resistance: twice weekly	Aerobic: RPE 12–13 Resistance: 3 sets, 8–12 reps, RPE 12–13	Aerobic: 20 mins Resistance: 30 mins	Median: 17 weeks (5–35 weeks)	Aerobic (walking, cycling, elliptical) and resistance (25 exercises (8 per session), bands, upper and lower muscle groups)	Unsupervised, home-based	Resistance: increase resistance when 3 × 12 performed without difficulty	No adverse events reported
Ngo-Huang et al. (2019)	Aerobic: 3x/week Resistance: twice weekly	Aerobic: RPE 12–13 Resistance: 3 sets, 8–12 reps, RPE 12–13	Aerobic: 20 mins Resistance: 30 mins	Mean: 16 ± 9	Aerobic (walking, cycling, elliptical) and resistance (25 exercises (8 per session), bands, upper and lower muscle groups)	Unsupervised, home-based	Resistance: increase resistance when 3 × 12 performed without difficulty	No adverse events reported
Niels et al. (2018)	Twice weekly	Aerobic: 70–80% HRmax, RPE 6–7 (Borg 0–10) Resistance: 2 sets, 8–12 reps, 70–80% 1-RM	Aerobic: 4–10 min	7 months	Aerobic (cycling and cross-trainer) and resistance (6 exercises, machine based, upper and lower muscle groups)	Supervised, location not specified	No progression described	No adverse events reported
Steindorf et al. (2019)	Twice weekly	Supervised: 1–3 sets, 8–20 reps, 50–80% 1-RM Unsupervised: 1–3 sets, 8–20 reps, RPE 14–16 (Borg 6–20)	60 mins	6 months	Resistance exercise Supervised: 8 exercises machine based, upper and lower muscle groups Unsupervised: 8 exercises, bands and dumbbells, upper and lower muscle groups	Supervised: university exercise facility Unsupervised: home-based	Both Supervised and unsupervised: 4 week adaptation period (5 exercise, 1–2 sets, 20 reps, 50–60% 1-RM. From week 5, 8 exercises, 2–3 sets, 8–12 reps, 60–80% 1-RM. Supervised: Progressive increase in resistance (5%) after successful completion of 3 sets, 12 reps, 3 consecutive sessions.	No adverse events reported
Wiskemann et al. (2019)	Twice weekly	Supervised: 1–3 sets, 8–20 reps, 50–80% 1-RM Unsupervised: 1–3 sets, 8–20 reps, RPE 14–16 (Borg 6–20)	60 mins	6 months	Resistance exercise Supervised: 8 exercises machine based, upper and lower muscle groups Unsupervised: 8 exercises, bands and dumbbells, upper and lower muscle groups	Supervised: university exercise facility Unsupervised: home-based	Both Supervised and unsupervised: 4 week adaptation period (5 exercise, 1–2 sets, 20 reps, 50–60% 1-RM. From week 5, 8 exercises, 2–3 sets, 8–12 reps, 60–80% 1-RM. Supervised: Progressive increase in resistance (5%) after successful completion of 3 sets, 12 reps, 3 consecutive sessions.	No adverse events reported
Yeo et al. (2012)	Daily	Brisk, unspecified	10–30 min	3 months	Aerobic (walking)	Unsupervised: home-based,	week 1–4: 10 mins week 5–8: 20 mins week 9–12: 25–30 min	Not reported

### Quality assessment

Included studies were assessed independently by two reviewers (DO’C, GP) using the Effective Public Health Practice Project Quality Assessment Tool (EPHPP). The EPHPP assesses six domains: (1) Selection bias, (2) Study design. (3) Confounders, (4) Blinding, (5) Data collection method, and (6) withdrawals / drop-outs, and gives an overall methodological rating of strong (no weak ratings), moderate (one weak rating), or weak (two or more weak ratings). In addition, the PEDro scale was used to assess risk of bias in randomised controlled trials (RCTs). This 11 item scale rates the methodological quality of RCT’s, with points awarded to each of the 11 items if clearly satisfied https://pedro.org.au/wp-content/uploads/PEDro_scale.pdf. Disagreements were resolved by consensus.

### Data synthesis

A narrative approach to analysis was proposed, summarising all included studies and extracting outcomes of interest to present a descriptive synthesis of important study characteristics. Secondary outcomes of exercise prescription were also summarised narratively.

## Results

### Study selection and characteristics

**Fig. 1:**
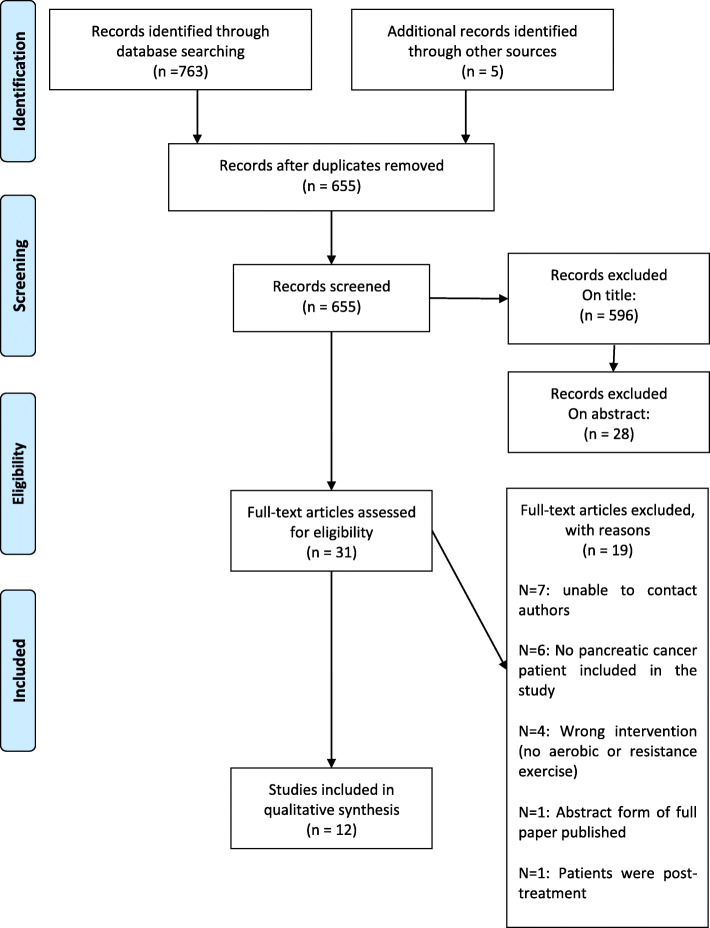
PRISMA flow diagram of the study selection process

The study selection process is detailed in Fig. 1. A total of 768 studies were identified from the databases. Twelve full text articles were included for final analysis: three RCT’s [16–18] five single arm prospective trials [19–23], three case reports [24–26] and one case series [27]. In the three RCT’s, one study involved participants being randomised to an exercise intervention or control group [16], and two studies involved participants being randomised to a supervised exercise group, an unsupervised exercise group, or control group [17, 18]. Of the twelve included studies, 9 (75%) were published between 2017 and present, and the remaining studies were published in 2012 [16] and 2014 [21, 24] respectively. Authors were contacted for information for seven additional studies identified which included participants with a pancreatic cancer diagnosis [28–34]. However, following three attempts to contact corresponding authors, all seven studies were excluded when no response was received. Heterogeneity of study types and the differing outcome measures within eliminated the possibility of conducting a meta-analysis.

### Risk of bias

Within the three RCT’s included [16–18], the mean PEDro score was 6.3 (0.6 SD). When all included studies were evaluated using the EPHPP tool, one of the twelve studies received a strong global rating [16], two a moderate rating [17, 18] and nine were rated as weak [19–27]. Limitations in weak study methodologies included study design, lack of blinding and lack of reporting of confounders.

### Study setting

Three of the 12 studies included prehabilitation exercise and had participants exercise in the period between diagnosis and surgery [19, 20, 27] and eight studies included rehabilitation exercise, with 5 of the 8 studies implementing exercise in participants who had undergone surgery and begun adjuvant therapy [16–18, 21, 24] and two studies which implemented exercise in participants with advanced pancreatic cancer undergoing palliative chemotherapy [22, 23]. One study implemented exercise in an individual with inoperable disease [26]. One study included an exercise intervention delivered during palliative chemotherapy, neo-adjuvant chemotherapy and following surgery during adjuvant chemotherapy [25]. McLaughlin et al., [26] in their case report had their participant complete 40 min of aerobic exercise on a cycle ergometer at 60% heart rate maximum (HR_max_) during chemotherapy infusions.

### Participant demographics

Analyzed sample sizes ranged from 1 to 102 participants, resulting in a total of 300 participants from twelve studies included in this review. Of these, 157 (52%) were male, and 143 (48%) were female. The mean age of participants included in this review ranged from 46 [25] to 74 years [27]. The study by Banzer et al., [21] involved a heterogenous group of cancer patients (*n* = 101), which included three pancreatic cancer patients. The studies by Mouri et al. [23] and Naito et al., [22] involved participants with advanced pancreatic (*n* = 6) and non-small cell lung cancer (NSCLC). Data for these participants are individually reported.

## Exercise prescription in pancreatic cancer

### Exercise type

Of the twelve included studies, two studies focused on aerobic exercise only [16, 21], four studies focused on resistance training only [17, 18, 22, 23], and six studies included a combination of aerobic and resistance exercise [19, 20, 24–27]. Six studies used unsupervised home-based exercise [16, 19–23], four studies used supervised exercise [24–27] with locations reported in two studies which included an exercise clinic [24] and hospital [26]. Two studies used supervised (university exercise facility) and home-based, unsupervised exercise [17, 18]. Exercise sessions were supervised by an exercise physiologist in two studies [24, 27]. One study reported supervision from the study researcher but did not allude to their professional background [26]. One study did not report who supervised the sessions [25]. Supervised exercise sessions were one-to-one, whilst one study progressed from two one-to-one sessions, to group exercise [24]. One study offered supervised exercise (Nordic walking, 1x/week, 60 mins) in addition to the study intervention [21].

### Intervention length

Exercise interventions delivered during the prehabilitation phase lasted the length of preoperative therapy [19, 20, 27]. Ngo-Huang et al., [20] in their 2017 study reported a mean intervention length of 16 weeks, whilst during their 2019 study reported a median intervention length of 17 weeks [19]. Marker et al., [27] in their case series of three participants, reported two participants with intervention lengths of 17 and 21 weeks. Studies conducted in the post-operative period were typically longer and ranged from 8 weeks to 6 months [16–18, 21–24, 26]. Niels et al., [25] started with a 3 month intervention during palliative chemotherapy, followed by a 4-month post-surgery intervention, delivered in parallel to adjuvant therapy.

### Intervention adherence

Six studies reported adherence to the exercise intervention which ranged from 64% [17] to 94% [26]. Wiskemann et al., [17] reported lower adherence to supervised resistance exercise (RT1) vs home-based resistance exercise (RT2) (64.1% Vs 78.4%). Steindorf et al., [18] reported a decrease in resistance exercise adherence over a 6 month intervention period for both supervised (73.6 to 41.5%) and home-based (87.5 to 62.0%) groups. Ngo-Huang et al., [20] reported better adherence to the aerobic exercise (12/15 met or exceeded recommendations) component of their intervention versus resistance exercise (6/15 met or exceeded recommendations).

## FITT-VP prescription and progression of exercise

Table 2 details the prescription of exercise in pancreatic cancer under the heading’s frequency, intensity, time, type, volume and documents patterns of progression where reported. A lack of consensus between studies is clear.

### Frequency

Where supervised exercise sessions were provided, the frequency of sessions was twice per week, [17, 18, 24–26]. One study documented an aerobic exercise session frequency of 3 x/week [20]. Three studies reported frequency ranges of 3–5 x/week [16, 21] and 2–3 x/week [27] respectively. One study did not specify an aerobic exercise session frequency [19]. Resistance exercise frequency was reported as 2x/week in seven studies [17–20, 24–26]. Two studies reported daily, home-based resistance training [22, 23]. Two studies advised participants to try and achieve, in addition to the exercise intervention, self-guided aerobic exercise for 150 mins/week [24, 26].

### Intensity

Aerobic exercise intensity was prescribed using % of HR_max_ and rating of perceived exertion (RPE). Resistance exercise intensity was prescribed using % of hypothetical 1-RM (h1RM), % of 1-RM and RPE. For studies prescribing aerobic exercise intensity using HR_max_, intensity ranged from 65 to 80% [24–26]. Cormie et al., [24] prescribed aerobic exercise using both HR_max_ (65–80%) and RPE (11–13, Borg 6–20). Banzer et al., [21] prescribed an aerobic exercise intensity of 13–14 RPE, whilst Huang et al., [20] used an RPE of 12–13 for both aerobic and resistance exercise intensity. Yeo et al., [16] prescribed brisk walking. Wiskemann et al., [17] and Steindorf et al., [18] prescribed supervised resistance exercise at an intensity of 50–80% 1-RM, and unsupervised exercise at 11–16 RPE. Mouri et al., [23] and Naito et al., [22] prescribed ‘low intensity’ resistance training. McLaughlin et al., [26] prescribed resistance exercise at 60% 1-RM, whilst Cormie et al., [24] prescribed 12-RM intensity. Niels et al., [25] prescribed their resistance exercise component at 70–80% h1RM. One study did not specify aerobic or resistance exercise intensity [19].

### Time / duration

Aerobic exercise was described in six studies, with the length of aerobic exercise sessions ranging from 10 mins to 45 mins [16, 20, 21, 24–26]. Due to the nature of the exercise performed, the duration of resistance exercise sessions was not always easy to determine. Six studies reported sets / repetitions, ranging from 1 to 4 sets, and 6–20 repetitions [17, 18, 20, 22–26]. One study reported resistance exercise sessions lasting 60 min [19], while another reported a combined aerobic / resistance exercise session length of 60 min [27].

### Type

Aerobic exercise modalities included walking [16, 19, 20] and cycle ergometry [24–26]. One study allowed participants to choose between treadmill walking, elliptical, rowing machine or recumbent bike [27], whilst one study allowed participants to choose from hiking, walking, running, cycling or swimming based on their preferences [21]. Resistance exercise modalities included resistance bands [19, 20], resistance exercise machines [17, 18, 24–26], free weights [17, 18] and body weight exercises [22, 23]. Two studies used resistance machines during supervised exercise, and resistance bands and free weights during unsupervised, home-based resistance exercise [17, 18].

### Volume

Weekly exercise volume was reported in five studies and ranged from 90 to 180 mins per week [16–20]. One study recommended a weekly aerobic exercise volume of 6–240 min [21]. Six studies did not specify weekly exercise volume [22–27].

### Progression

Progression of the exercise prescription was reported in nine of the twelve studies [16–24]. Wiskemann et al., [17] and Steindorf et al., [18] reported on the same exercise intervention. For the supervised exercise group, after a 4-week adaptation period (5 exercises, 1–2 sets, 20 repetitions, 50–60% 1-RM), participants completed 8 exercises for 3 sets and 8–12 repetitions at 60–80% 1-RM. Weight lifted was progressed by 5% for each exercise after completion of 3 sets of 12 repetitions for 3 consecutive sessions. The unsupervised, home-based group following a 4-week adaptation period (5 exercises, 1–2 sets, 20 repetitions at a low – moderate intensity (RPE 11–13)), progressed to 8 exercises of 3 sets, and 8–12 repetitions at intensity of RPE 14–16. Yeo et al., [16] had participants progress from 10 min of brisk walking during month 1 of their walking programme to 30 min during month 3. Ngo-Huang et al., [19, 20] progressed the resistance exercise component of their exercise intervention. Participants completed 8 exercises per session with participants progressing to a new exercise once the highest resistance band was used. Niels et al., [25] in their case report had their participant progress the resistance exercise component to include eccentric resistance with 30% of the concentric h1-RM when the participant moved from palliative chemotherapy to neo-adjuvant chemotherapy in preparation for surgery. Cormie et al., [24] reported increasing resistance for upper and lower body exercises during the next session if the participant worked beyond the RM target the previous session. The level of increment was not specified. Mouri et al., [23] and Naito et al., [22] reported on the same intervention, and following assessment of participant performance and tolerability to the home exercise intervention through data from exercise diaries and direct interviews, made adjustments to the exercise programme; however, no further details are provided. One study reported that after 4-weeks, home-based exercise was adjusted to condition, side effect status and exercise preferences, but no further details were provided [21].

### Inclusion of control group

Three studies included control groups. In two of the RCT’s [17, 18], the control group received usual care without the exercise intervention and were contacted by the exercise specialist by telephone monthly to ask about cancer treatment adverse effects. During chemotherapy, the control group was offered nutrition and psychosocial counselling. One study had the control group receive usual care without the exercise intervention and no monthly phone calls. They were encouraged at the start of the trial to perform usual activity / exercise [16].

## Exercise safety in pancreatic cancer

Nine studies reported no serious adverse events when participants underwent exercise both prior to surgery or following surgery [17–20, 22–26]. Three studies did not report serious adverse events as an outcome [16, 21, 27].

## Effects of exercise on body function and structure

### Exercise capacity

Exercise capacity was directly (VO_2_peak) assessed using cardiopulmonary exercise testing (CPET) in two studies [17, 21], estimated (VO_2_max) in one study using the Astrand Rhyming cycle ergometer test [26], one study using a modified WHO cycle ergometer test which was also completed on a cross trainer measured exercise performance (measured in Watts) [25], three studies used the 6-min walk test (6MWT) [17, 19, 22], two studies used the 400-m walk test [24, 27], three studies implemented the stair climb test [24, 26, 27] and one study used self-reported walking distance [16]. Wiskemann et al., [17] in a three group RCT comparing supervised resistance exercise (RT1), unsupervised resistance exercise (RT2) and a no exercise control group (CON) reported no significant differences between groups (RT1 vs CON, *p* = 0.43; RT2 vs CON, *p* = 0.22; RT1 vs RT2, *p* = 0.79) for peak oxygen uptake (VO_2peak_). The authors did report significant differences for peak work rate between RT1 and CON (*p* = 0.03) and RT1 and RT2 (*p* = 0.03) in favor of RT1. Banzer et al., [21] in their single arm prospective study, which included three participants with pancreatic cancer reported improvements of 5.7 and 8.7 ml/kg/min for two participants, and a decrease of 3.2 ml/kg/min in one participant after 16 weeks of home-based aerobic exercise. McLaughlin et al., [26] in a case report involving a participant with stage III locally advanced disease reported a ~ 9% improvement in estimated VO_2max_ after 12 weeks of a concurrent exercise intervention. A comparable case report involving a participant with stage IV pancreatic cancer reported a 50% improvement in aerobic capacity (90 W to 135 W) after 7 months of concurrent exercise [17].

Ngo-Huang et al., [19] in a single arm prospective trial reported a significant improvement in 6MWT after their pre-operative exercise intervention from baseline (463 m vs 488 m, *p* = 0.001). Wiskemann et al., [17] reported no change in 6MWT performance after their 6 month resistance exercise intervention from baseline for all groups, and no between group differences in mean change (RT1 vs CON, *p* = 0.42; RT2 vs CON, *p* = 0.64; RT1 vs RT2, *p* = 0.21). Naito et al., [22] reported a mean increase of 18 m in 6MWT performance after 8 weeks of home-based resistance training six participants. Cormie et al., [24] in a case report involving a participant with stage IIB pancreatic cancer reported at 17.5% improvement in time to complete the 400-m walk test after 6 months of concurrent exercise (204 s vs 247 s). The authors also reported a 19% improvement in the time to complete the stair climb test (3.92 s to 3.17 s). Marker et al., [27] in a case series involving three participants, reported 400-m walk test improvements in all three participants of 8, 23, and 24 s respectively. The authors also implemented the stair climb test, with two participants demonstrating a deterioration in performance (− 11% and − 21%), and one participant improving (+ 5%). McLaughlin et al., [26] reported a ~ 3% improvement in stair climb performance. Yeo et al., [16] in their RCT involving predominantly participants with stage IIA and IIB pancreatic cancer reported no significant difference between the intervention and usual care group for self-reported walking distance (10,772 vs 5219 ft., *p* = 0.1) at 12 week follow-up from baseline. However, a significant difference between groups was detected for the number of participants still self-reporting walking at the end of the study (intervention, 33/41; control, 18.31, *p* = 0.04).

### Muscle strength

Seven studies assessed muscle strength using diverse outcome measures [17, 19, 20, 24–27]. One study used one repetition maximum (1-RM) testing [24]. Niels et al., [25] used a hypothetical 1-RM (h1-RM), calculated using a validated formula to assess muscle strength. McLaughlin et al., [26] used 12-RM testing. Wiskemann et al., [17] measured isokinetic and isometric muscle strength via fixed and hand-held muscle dynamometry. Four studies assessed strength as measured using hand grip strength [19, 20, 22, 27]. Cormie et al., [24] reported a 42% (86 kg to 122 kg) improvement in leg press 1-RM after 6 months of concurrent exercise from baseline. Niels et al., [25] using a h1-RM (determined from 3 to 8 repetitions to exhaustion using a pre-defined weight), reported improvements after their 7 month concurrent exercise intervention from baseline in both upper (chest press, 43%; seated row, 3%; back extension, 63%) and lower body (leg extension, 79%) muscle strength, with the exception of leg curl (− 3.6%) and abdominal crunch (− 88%). In agreement, McLaughlin et al., [26] demonstrated improvements in both upper and lower body strength, as did Wiskemann and colleagues [17]. They analysed changes in maximal isokinetic peak torque and reported statistically significant differences between RT1 and CON and RT2 for elbow flexor (RT1 vs CON *p* = 0.02; RT1 vs RT2, *p* = 0.046) and extensor strength (RT1 vs CON *p* = 0.01; RT1 vs RT2, *p* = 0.03) [12]. Statistically significant differences for maximal voluntary isometric contraction were also reported between RT1 and CON for elbow flexor (*p* = 0.02) and knee extension (*p* = 0.01), and between RT2 and CON for knee extensor (*p* = 0.04). The authors, using hand-held dynamometry also reported statistically significant differences between RT1 and RT2 for knee flexor strength (*p* = 0. 01) and between RT2 and CON for knee extensor strength (*p* = 0.04).

Naito et al., [22] reported a mean decrease in handgrip strength of 2 kg of the dominant hand after 8 weeks of home-based resistance training. Marker et al., [27] reported improvements in handgrip strength of the dominant hand in two participants (+ 3% and + 2%), a deterioration in one participant (− 1%) and a decrease in strength in the non-dominant hand in all three participants (− 8%, − 7% and − 4%). Contrarily, Ngo-Huang et al., [20] in their single arm prospective trial reported no significant change in handgrip strength after the intervention from baseline (− 0.6 kg, *p* = 0.09). In a recent follow-up trial, Ngo-Huang et al., [19] reported no significant changes in handgrip strength from baseline to post intervention (0 kg, *p* = 0.9).

### Functional capacity

Six studies assessed functional muscle strength with six studies using the 5 x sit to stand test (5xSTS) [19, 20, 22, 24, 26], and one study using the 30 s sit to stand test (30STS) [27]. Ngo-Huang et al., [20] reported no significant change in 5xSTS performance (*p* = 1.0), but later detected a significant improvement in 5xSTS performance from baseline to post intervention (11.4 s to 10.6 s, *p* = 0.049) [14]. Naito et al., [22] reported a mean deterioration in 5xSTS performance of 21% (10.9 s vs 13.2 s). Cormie et al., [24] reported a 28% improvement in 5xSTS performance (11.38 s to 8.17 s), while McLaughlin et al., [26] showed a 9% improvement using the same measure. Marker et al., [27] reported improvements in 30STS performance in two participants of 54% (13 to 20 reps) and 44% (9 to 13), while the remaining patient prevented deterioration by maintaining performance (22 reps).

Five studies assessed gait speed using diverse outcome measures. Two used 10-m walk test [20, 27], two studies used the 6-m walk test [24, 26], one study used the 5-m walk test [22], and two studies used the 3-m walk test [19, 20]. Ngo-Huang et al., [20] reported no significant change in 10-m walk performance after their exercise intervention (6.61 s vs 5.93 s, *p* = 1.0). Marker et al., [27] reported mixed results across both usual and fast paced 10-m walk test performance. One participant improved (+ 7%, 1.5 m/s to 1.7 m/s), one participant deteriorated (− 6%, 1.8 m/s to 1.7 m/s) and one participant did not change (0%, 1.6 m/s to 1.6 m/s) usual paced 10-m walk performance. Two participants improved fast paced 10-m walk performance (+ 4%, 2.3 m/s to 2.4 m/s; + 9%, 2.2 m/s to 2.4 m/s) and one participant deteriorated (− 8%, 2.5 m/s to 2.3 m/s). Cormie et al., [24] reported a 27% and 21% improvement in usual and fast paced 6-m walk performance respectively. Consistently, McLaughlin et al., [26] reported a 15% improvement in 6-m walk performance, but did not specify for usual / fast / /backwards attempts. Naito et al., [22] reported a 6% change in 5-m walk (1.2 m/s to 1.13 m/s) performance in six participants. Ngo-Huang et al., [20] reported no change in 3-m walk performance across their intervention period (*p* = 1.0). Meanwhile, in their follow-up prospective trial, Ngo-Huang et al., [19] reported a significant improvement in 3-m walk performance post-intervention (1.17 m/s to 1.22 m/s, *p* = 0.009).

Two studies assessed static and dynamic balance. One study used the backwards 6-m walk test and the sensory organization test [24], with the other using the dynamic gait index [20]. Cormie et al., [24] reported a 5% improvement in static balance and a 23% improvement in dynamic balance. Ngo-Huang et al., [20] reported no significant improvement in the dynamic gait index across their intervention period (*p* = 0.65).

### Body composition

Dual Energy X-ray absorptiometry (DXA) was used to assess whole body fat mass and lean mass in two studies [24, 27]. One study used bioelectrical impedance analysis (BIA) to assess percentage body fat and lean mass [26]. Cormie et al., [24] showed that concurrent exercise can led to an increase in both whole body lean mass (+ 3%, 62.9 to 65.0 kg) and appendicular lean mass (+ 8%, 26.8 to 29.0 kg). The authors also reported an increase in whole body fat mass (+ 2%, 36.8 to 37.5 kg). Marker et al., [27] reported improvements all three participants in whole body lean mass (+ 15%, 49.8 to 57.0 kg; + 1%, 57.9 to 58.4 kg; + 4%, 37.2 to 38.9 kg) and appendicular lean mass (+ 18%, 21.7 to 25.6 kg; + 7%, 23.9 to 25.6 kg; + 3%, 15.1 to 15.5 kg). Over the same period, the authors reported an improvement in whole body fat mass in two participants (+ 9%, 11.2 to 12.2 kg; + 6%, 6.2 to 6.6 kg) and deterioration in one participant (− 15%, 18.1 to 15.3 kg). McLaughlin et al., [26] in their case report also reported improved percentage body fat (− 25%, 17.7 to 13.3 kg) and lean mass (+ 5%, 82.3 to 86.7 kg).

## Effects of exercise on fatigue and health related quality of life

### Fatigue

Six studies assessed cancer-related fatigue [16, 18, 24–27]. Six studies utilized multidimensional fatigue scales; four studies used the Functional Assessment of Chronic Illness Therapy-Fatigue subscale (FACIT-F) [16, 24, 26, 27], two studies used the Multi-dimensional Fatigue Inventory (MFI) [18, 25], and one study used a unidimensional Fatigue visual numeric rating scale (VAS) [16]. Yeo et al., [16] reported significant improvements in FACIT-F (27 to 36, *p* = 0.05) and VAS (4.8 to 3.5, p = 0.05) scales in the experimental group post-intervention, with no changes detected at the same time points in the usual care group. Cormie et al., [24] reported an improvement in FACIT-F subscale from 8 to 47 after 6 months of concurrent exercise in their case report. Similarly in another case report, McLaughlin et al., [26] showed a 110% improvement at week 12, using the FACIT-F subscale. Marker et al., [27] in their case series reported inconsistent FACIT-F subscale scores after the intervention, with one patient improving (40 to 50), one patient remaining the same as baseline (36) and the remaining patient failed to complete reassessment.

Steindorf et al., [18] in their RCT reported no significant differences in any MFI subscale score between groups at 6 months. Niels et al., [25] in their case reported stability in MFI scores across the intervention except for dimensions general (4 to 5) and physical (4 to 5) fatigue and reduced motivation (4 to 5).

### Quality of life

Eight studies assessed health related quality of life [16, 18, 19, 23–26]. Three studies used the Functional Assessment of Cancer Therapy - Hepatobiliary Cancer (FACT-Hep) [19, 24, 26], four studies used the European Organization for Research and Treatment of Cancer Quality of Life Core 30 questionnaire (EORTC QLQ-C30) [18, 21, 23, 25], two studies used the FACT-G [19, 27] one study used the EORTC QLQ pancreatic specific module (EORTC PAN26) [18] and one study used the Short Form-36v2 health survey (SF-36v2) [16]. Ngo-Huang et al., [19] in their single arm prospective study reported no significant change in FACT-Hep (137.9 to 142.3, *p* = 0.09) and FACT-G score (84.0 to 85.5, p = 0.09). Cormie et al., [24] reported improvements in FACT-Hep score (115 to 152). McLaughlin et al., [26] reported a 38% improvement in FACT-Hep score after 12 weeks of concurrent exercise.

Niels et al., [25] reported improvements in 5 of 6 function subscales of the EORTC QLQ-C30 (Global health score, Physical, Role Emotional and Social), and improvements in 3 of 9 symptom subscales (Fatigue, Nausea / vomiting, and Pain). Banzer et al., [21] reported improvements of 17 and 25 points in Global health score in two of three participants. In addition, they reported improvement in Fatigue symptom subscale of 11 points in two participants and 22 points in one participant. Mouri et al., [23] reported a deterioration in Global QoL score and Physical QoL subscale of 1 point and 7 points respectively after 8 weeks of home-based resistance training.

Steindorf et al., [18] reported no significant group differences across all EORTC QLQ C-30 and EORTC PAN26 subscales between supervised and unsupervised resistance exercise groups and control group at 6 months (*p* = 0.93) but when resistance exercise groups were pooled, a significant group difference was observed between resistance exercise and control at 3 months for Global Quality of Life (*p* = 0.016), Physical functioning subscale (p = 0.016), Cognitive functioning subscale (*p* = 0.008) and sleep problem (p = 0.016). Marker et al., showed an increased FACT-G score in 2 of 3 participants (81 to 97; 83 to 100), with their remaining patient not completing post-intervention reassessment.

## Discussion

The aim of this systematic review was to summarise the available evidence regarding the efficacy and prescription of exercise in individuals undergoing treatment for pancreatic cancer and to provide a comprehensive overview, not limited by study type. The findings of this review suggest that when compared to usual care, exercise in both a pre-surgical, during neo-adjuvant therapy (prehabilitation), and post-surgery adjuvant therapy context (rehabilitation), is safe with no serious adverse events reported. The results illustrate the infancy of the area, with only ten studies meeting the criteria for analysis. Of the studies included, only three were RCT’s. The exercise interventions and outcome measures selected in the included studies varied considerably preventing a meta-analysis. This lack of consensus regarding the most appropriate exercise prescription and outcome measures to assess intervention effectiveness in this population hampers the interpretation of the current evidence. As such, this highlights the need for well conducted prospective trials to assess the impact of exercise programmes on outcomes and QOL in pancreatic cancer patients.

The quality of the studies included was variable, with the highest level of evidence drawn from three RCT’s [16–18]. Methodological quality, assessed via the PEDro scale, suggests these studies were of moderate to high-quality. However, the findings should be interpreted with caution, given limitations were identified in sample size and blinding procedures. The remaining studies included prospective studies (*n* = 3), a case series and case reports (n = 3) which despite promising results for the efficacy of exercise in pancreatic cancer, provide lower levels of evidence. When all studies were assessed using the EPHPP, the majority of studies (67%) received a weak global rating for quality. As such, further adequately powered RCT’s, which use validated and appropriate outcome measures, and blinding of outcome assessors are required.

The current evidence seems to suggest that exercise is associated with improvements in muscle strength, functional capacity, body composition, fatigue and quality of life across pancreatic cancer treatment regimens. These results should, however, be interpreted with some caution. Improvements in outcomes were observed in some, but not all studies. Variability is likely related to differences in study design, outcome measures and sample size. Recruitment difficulties were reported in two of the RCT’s [17, 18] and therefore further work assessing the feasibility of conducting larger RCT in this patient population is required. Furthermore, the studies varied substantially with regards to the nature of the interventions implemented (e.g. exercise timing, intensity, duration, modality). This heterogeneity may be linked to the variable rates of exercise adherence which were reported across studies. As such, it is currently not possible to determine the optimal (or minimally acceptable) dose of exercise nor the optimal intervention delivery methods for this population, to induce the desired health outcomes.

Exercise capacity is an important component of physical fitness and has been linked to higher chemotherapy completion and lower complications rates in breast and colon cancer patients [35, 36]. VO_2peak_ levels in pancreatic cancer patients are reported as 18–24% below normative reference values [37] highlighting a key physiological target for interventions. However, only two studies included in this review directly assessed VO_2peak_. Wiskemann et al., [17] in their RCT implemented a resistance exercise intervention over a 6 month period and reported no significant change in VO_2peak_ as measured via CPET. When considered in the context of training specificity, this result is unsurprising given the nature of the intervention used. The use of walking-based tests over gold standard assessments (CPET) in the majority of included studies (56%) highlights the clinical relevance of these tests in this population, due to walking being central to activities of daily living and provides support for their use in future trials.

Sarcopenia, the age related decrease in muscle mass and function is common amongst individuals with PDAC [37], and is associated with poorer overall survival [38]. Indeed, reduced muscle strength values for the lower limb muscles of 12–15% below healthy reference values have been reported [39]. Muscle strength and body composition (whole body lean mass) was improved in studies which included a resistance exercise component [24, 26, 27]. The interventions reporting strength and body composition improvements employed a resistance exercise prescription during the rehabilitation phase which complies with current exercise recommendations for other cancer populations [12]. However, due to limitations in study design (case reports / case series) the evidence is not sufficient to make recommendations on resistance exercise FITT prescription in pancreatic cancer to enhance strength and body composition.

The majority of studies reported improvements in quality of life and fatigue with exercise training. Steindorf et al., [18] reported clinically meaningful improvements in quality of life and physical fatigue with significant differences between the supervised and unsupervised resistance exercise groups and control at 3 months. However, after 6 months no differences were reported. An explanation for this likely multifactorial, but a component of this may be the steady decline in exercise adherence which was reported for both supervised (74–42%) and unsupervised (88–62%) resistance exercise groups over the 6-month period. However, the difference at 3-months between exercise groups is clinically relevant, and highlights the need for more well-designed prospective trials investigating the utility of supervised exercise in individuals with PDAC.

Although the exercise prescription varied between studies, the majority (67%) included in this review complied with current exercise recommendations in oncology (frequency, intensity, time). When looking at the timing of the delivery of the intervention, three studies occurred between diagnosis and surgery and four studies occurred following surgery, during adjuvant chemotherapy. Interestingly, McLaughlin et al., [26] encouraged their participant, who entered the study with inoperable disease, to exercise during chemotherapy infusions. Tumour response (reduced tumour mass) to cancer treatment was reported after 6 cycles of chemotherapy resulting in a successful Whipple procedure. Exercise during chemotherapy infusions has been linked to increased treatment efficacy [40]. Proposed mechanisms include increased treatment tolerance (due to exercise increasing blood flow to skeletal muscle, away from splanchnic organs) and increased tumour blood flow (+ 200%), tumour perfusion and delivery of chemotherapy [41]. Indeed, a recent study has demonstrated improved tumour vasculature in response to 14 weeks of pre-operative aerobic and resistance exercise in pancreatic cancer patients [42]. Together, this highlights an exciting area of research which warrants further investigation.

Adherence to exercise interventions during cancer treatment can be poor [43]. This was evident in the studies included in this review, where adherence to exercise was variable (64–94%). In this regard, the results presented by Wiskemann et al., [17] showed better adherence to unsupervised resistance exercise versus supervised resistance exercise. This difference may be founded in the cited barriers to exercise within the supervised group. Barriers including lack of time, competing medical appointments and travel distance, all feature in the pancreatic cancer literature [44]. However, despite poorer adherence, participants in the supervised group achieved greater gains in muscle strength. The reasons for this may be three-fold; greater motivation to exercise and a higher exercise intensity achieved during supervised sessions, and/ or biased self-reporting of adherence in unsupervised groups. Supervised exercise sessions are linked to greater improvements in health outcomes in cancer patients [45]. However, with developments in digital technology, a desire for unsupervised, home-based exercise in some pancreatic cancer patients [44], and the necessity for home-based exercise due to current restrictions imposed by the coronavirus (Covid-19) pandemic, future studies should now consider more effective and safe methods of remotely monitoring exercise intensity and adherence, where unsupervised exercise is implemented.

Presently, there is a paucity of qualitative evidence in the pancreatic cancer and exercise literature. Expanding this evidence base and incorporating the opinions of patients and healthcare professionals in study design and exercise intervention development / feasibility would be advantageous. Healthcare intervention development relies on feedback from multiple stakeholders, and patient and public involvement (PPI) in the development phase may lead to a more effective intervention [46]. This highlights an area for future work which may help ensure adequately designed studies and appropriately developed interventions.

## Conclusions

The scientific literature investigating the effects exercise in pancreatic cancer patients undergoing treatment is sparse and limited by a lack of high quality, adequately powered RCT’s. Existing evidence is suggestive of exercise as an effective intervention to help mitigate common disease / treatment complications including impaired physical function, quality of life and fatigue. However, there is insufficient evidence to conclude the optimal timing and design of exercise programming for individuals undergoing treatment for pancreatic cancer. Future studies should include input from key stakeholders in the intervention design and development phase to ensure appropriately designed and developed studies.

## Supplementary Information

**Additional file 1.**

## Data Availability

All data generated or used during the study appear in the submitted article.

## Abbreviations

6MWT: 6 min walk test
BIA: Bioelectrical impedance analysis
DXA: Dual Energy X-ray absorptiometry
EORTC QLQ-C30: European Organization for Research and Treatment of Cancer Quality of Life Core 30 questionnaire
EORTC PAN26: European Organization for Research and Treatment of Cancer Quality of Life pancreatic specific module
EPHPP: Effective Public Health Practice Project Quality Assessment Tool
FACIT -F: Functional Assessment of Chronic Illness Therapy-Fatigue subscale
FACT-Hep: Functional Assessment of Cancer Therapy - Hepatobiliary Cancer
FITT-VP: Frequency, intensity, time, type, volume, progression
HR-QoL: Health related quality of life
MFI: Multi-dimensional Fatigue Inventory
m/s: Meters per second
NSCLC: Non-small cell lung cancer
PDAC: Pancreatic ductal adenocarcinoma
RCT: Randomised controlled trial
RM: Repetition maximum
RPE: Rating of perceived exertion
SD: Standard deviation
VO2max: Maximum oxygen uptake
WHO: World health organisation
