# Supplementary material for: Exercise efficacy and prescription during treatment for pancreatic ductal adenocarcinoma: a systematic review
Source: BMC Cancer. 2021 Jan 9;21:43. doi: 10.1186/s12885-020-07733-0 (PMC7794639; doi:10.1186/s12885-020-07733-0)
Supplement: Supplementary file 1 — Additional file 1. [file 12885_2020_7733_MOESM1_ESM.docx]

|  | **Population** | **Intervention** | **outcome** | **result** |
| --- | --- | --- | --- | --- |
| MEDLINE | exp Pancreatic neoplasms or carcinoma, pancreatic ductal or pancreas cancer.mp. or pancre* cancer.mp. or adenocarcinoma or chemotherapy, adjuvant or radiotherapy, adjuvant or drug therapy | Exercise or exercise therapy or resistance training or aerobic exercise.mp. or rehabilitation or prehabilitation.mp. or rehabilitation.mp. or exercise test or exercise test.mp. | Quality of life or physical fitness or muscle strength or cardiorespiratory fitness or body composition or physical function.mp. or musc* strength | Population = 303739  Intervention = 489412  Outcome = 292640  Combined = 221 |
| EMBASE | Pancreas cancer or pancreatic neoplasm.mp. or pancreas* cancer.mp. or adjuvant chemotherapy or adjuvant radiotherapy or cancer adjuvant therapy or adenocarcinoma or pancreatic ductal adenocarcinoma.mp. or pancreas adenocarcinoma | Exercise or aerobic exercise or exercise test or cardiopulmonary exercise test or muscle exercise or dynamic exercise or exercise.mp. or resistance training or strength training.mp. or kinesiotherapy or cancer rehabilitation or rehabilitation.mp. or prehabilitation.mp. | Fitness or physical fitness or quality of life or muscle strength or cardiorespiratory fitness or aerobic capacity or physical function.mp. or body composition or musc* strength.mp. or muscle atrophy | Population = 318670  Intervention = 434602  Outcome = 651797  Combined = 456 |
| CINAHL | Pancreatic neoplasms or pancreas cancer or pancreatic ductal adenocarcinoma or adenocarcinoma or pancreas* cancer or radiotherapy, adjuvant or chemotherapy, adjuvant | Exercise or exercise (MH) or resistance training or therapeutic exercise or aerobic exercises or muscle strengthening or exercise test, cardiopulmonary or exercise test, muscular or exercise test or rehabilitation or rehabilitation, cancer or prehabilitation | Physical fitness (MH) or physical fitness or physical endurance or physical function or quality of life or aerobic capacity or muscle strength or cardiorespiratory fitness or body composition or muscular atrophy | Population = 39033  Intervention = 175753  Outcome = 159546  Combined = 86 |
